# Study on the Voltage Reference Noise at Sub-Millihertz Frequencies for Developing an Ultra-Stable Temperature Measurement Subsystem

**DOI:** 10.3390/s23104611

**Published:** 2023-05-10

**Authors:** Lingyun Gu, Houyuan Chen, Peng Liu, Mingxuan Wen, Chen Ling, Zening Sun, Yanwei Ding

**Affiliations:** 1 School of Physics and Astronomy, Sun Yat-sen University, Zhuhai 519082, China; 2 DFH Satellite Co., Ltd., Beijing 100094, China

**Keywords:** ultra-low-frequency noise, voltage reference, noise budget, chopper amplification, two-channel measurement, thermal insulation

## Abstract

A temperature measurement subsystem (TMS) is a critical piece of infrastructure of the space gravitational wave detection platform, necessary for monitoring minuscule temperature changes at the level of $1\;\mu K/{\mathrm{Hz}}^{1/2}$ within the electrode house, in the frequency range of 0.1mHz to 1Hz. The voltage reference (VR), a key component of the TMS, must possess low noise characteristics in the detection band to minimize the impact on temperature measurements. However, the noise characteristics of the voltage reference in the sub-millihertz range have not been documented yet and require further study. This paper reports a dual-channel measurement method for measuring the low-frequency noise of VR chips down to 0.1mHz. The measurement method makes use of a dual-channel chopper amplifier and an assembly thermal insulation box to achieve a normalized resolution of $3\times 10^{-7}\;/{\mathrm{Hz}}^{1/2}\;@\;0.1\;\mathrm{mHz}$ in the VR noise measurement. The seven best-performance VR chips documented at a common frequency range are tested. The results show that their noise at sub-millihertz frequencies can significantly differ from that around 1Hz.

## 1. Introduction

In astronomy, gravitational waves (GWs) have been considered powerful messengers to probe the densest objects in space and discover the nature of the universe. The in-spiral orbiting of compact binaries, which may consist of white dwarfs, neutron stars, and even black holes, can emit GW signals that carry huge amounts of energy [1,2,3,4]. However, the classic signals, which merely distort our local frames of spacetime by an utterly minuscule amount, are hard to detect from a distance. The frequency lower bound of detectability for terrestrial detectors like LIGO and VIRGO is ∼10 Hz, limited by ground vibrations and the length of the interferometer baseline [5,6,7]. To increase the chances of detecting GW events in the millihertz frequency range, the concept of space-based GW detectors has been proposed [8].

The current generation of space-based detectors are constellation interferometers that consist of multiple satellites [9,10,11,12]. The GW signal can be evaluated by measuring the relative distance variation in arm lengths by inter-satellite laser interference. Drag-free control is applied to the satellites, which means they will follow their internal test mass (TM) to offset against non-gravitational forces. As the inertial reference for the measurement, the TM should be well-shielded, to maintain a high degree of geodesic trajectory. However, temperature fluctuations can induce residual acceleration to the TM through several different effects. Analysis shows that temperature variations of the electrode house, which contains the TM, must not exceed $10\;\mu K/{\mathrm{Hz}}^{1/2}$ in the ultra-low-frequency (ULF) band of 0.1 mHz–1 Hz [10,11,12,13].

The temperature measurement subsystem (TMS) is used to measure extremely small amounts of temperature change. It is generally required to have noise one order of magnitude smaller than the measurement target. Thus, to monitor the $10\;\mu K/{\mathrm{Hz}}^{1/2}$ temperature variation, the subsystem must have a noise of less than $1\;\mu K/{\mathrm{Hz}}^{1/2}$ in the ULF band.

According to the preliminary assignment, the noise-equivalent temperature (NET) contribution from the voltage reference (VR) noise must not exceed $0.57\;\mu K/{\mathrm{Hz}}^{1/2}$. This means that the upper limit of the normalized voltage noise for the VR chip is $2.8\times 10^{-5}\;/{\mathrm{Hz}}^{1/2}$. Most manufacturers would document the noise profiles of their VR chip products in the frequency range of 0.1–10 Hz. However, the required noise at sub-millihertz frequencies remains largely unknown from both the aspects of experimental tests and theoretical study [14,15,16]. Previous studies have focused on higher-frequency noise. For instance, ref. [17] introduced a low-noise VR that enhances the resolution of the capacitive accelerometer, with noise at 3 Hz as the primary concern. Similarly, ref. [18] demonstrated a low-noise bandgap VR and evaluated its noise, but only down to 1 Hz. Although ref. [19] evaluates the noise level of the VR close to DC, it relies on the simulation result. Therefore, a comprehensive study of the ULF noise of VRs is necessary.

State-of-the-art multimeters on the market are still not capable of directly measuring the VR noise at ULF [20]. The noise must be pre-amplified to reach a proper level. However, the conventional amplification method inevitably introduces the 1/f noise, which increases rapidly with the decrease in frequency [21]. It would overwhelm the VR noise to be measured in the ULF band. In addition, temperature fluctuations are another factor that affects the measurement. Temperature-induced noise at ULF can be confused with VR noise.

To this end, this paper presents a dual-channel chopper amplification method to mitigate the 1/f noise, and an insulated environment is established to suppress the ULF ambient temperature disturbances. The method can significantly improve the system’s measurement capability in the ULF band. Seven VR chips of low noise are selected from hundreds of other candidates based on the manufacturing data sheets. The voltage noise at their output pins is measured by the test system.

This paper is organized as follows. The temperature measurement scheme and the noise budget of each component are described in Section 2. Section 3 describes the VR noise measurement setup, including the measurement method, circuit design, insulation environment building, and system setup, and evaluates the system measurement capability. Section 4 selects seven low-noise VR chips for measurement. Section 5 measures the ULF noise of the selected seven VR chips, and the chips that meet the requirements are selected. The conclusions of this paper are presented in Section 6.

## 2. TMS’s Composition and Noise Budget

Electrical temperature measurements may use platinum resistors [22] or negative temperature coefficient (NTC) thermistors as temperature sensors [23]. The resistance of platinum resistors has good linearity with temperature, resulting in high measurement accuracy. By contrast, the resistance of NTC thermistors has an exponential relation to the temperature, which can improve measurement sensitivity. Therefore, the NTC thermistor is chosen as the sensor. For a similar reason, the wiring structure of the two-wire bridge is preferred over the four-wire connection [24].

**Figure 1:**
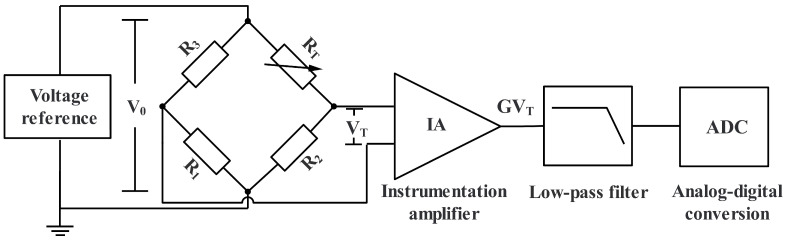
Composition of the temperature measurement subsystem.

The composition of our TMS includes a VR, a thermistor bridge, an amplifier, a filter, and an ADC, as shown in Figure 1. As a passive sensor, the NTC thermistor requires a stable external excitation $V_{0}$ to work. Since the output signal to be measured $V_{T}$ is feeble, amplification and filtering are required to improve the signal-to-noise ratio before it reaches the analog-to-digital conversion module.

Preliminary noise assignment for different parts of the TMS is carried out based on the target of $1\;\mu K/{\mathrm{Hz}}^{1/2}$. Before the measurement, as there is no available noise data to refer to in the ULF band, the noise budget is made in the way of equal-weight assignment, as shown in Table 1.

- Voltage reference

The role of the VR is to provide the excitation source for the NTC thermistor. Thus, the voltage source needs to be stable enough to reduce the self-heating noise caused by voltage fluctuations. The self-heating noise of the thermistor can be assessed by $N_{S}=\theta V_{0}N_{V}/\left(2R_{0}\right)$, which is related to the nominal voltage $V_{0}$, the voltage noise $N_{V}$, the thermistor nominal resistance $R_{0}$, and the thermal contact resistance θ [25]. Our preliminary consideration is $R_{0}=10\;k\Omega$, $V_{0}=2\;V$, and θ=100K/W. To stay within the noise budget of ≤$0.57\;\mu K/{\mathrm{Hz}}^{1/2}$, the normalized noise of the VR must satisfy $N_{V}/V_{0}\leq 2.8\times 10^{-5}\;/{\mathrm{Hz}}^{1/2}$ in the ULF band.

- NTC thermistor

The thermistor ($R_{T}$) and the three resistors ($R_{1}$, $R_{2}$, $R_{3}$) are wired to form a Wheatstone bridge circuit, whose output signal is sensitive to temperature. The balance point can be adjusted by changing the resistance values of the resistors. The output signal of the Wheatstone bridge can be expressed as:

(1) $$V_{T}=V_{0}\;\left(\frac{R_{T}}{R_{T}+R_{2}}-\frac{R_{3}}{R_{3}+R_{1}}\right),$$

where the NTC thermistor’s resistance has a temperature relation that $R_{T}=R_{0}\;e^{\beta (T^{-1}-T_{0}^{-1})}$.

According to noise synthesis theory [26], the output noise shall satisfy that:

(2) $$N_{V_{T}}^{2}={\left(\frac{\partial V_{T}}{\partial R_{T}}\right)}^{2}\;N_{R_{T}}^{2}+\sum_{n=1}^{3}{\left(\frac{\partial V_{T}}{\partial R_{n}}\right)}^{2}\;N_{R_{n}}^{2},$$

where the bridge noise $N_{V_{T}}^{2}$ is related to the thermistor noise $N_{R_{T}}^{2}$ and resistor noise $N_{R_{n}}^{2}$.

At ULFs, the resistors are much more stable than the thermistor [27], such that $N_{R_{n}}\ll N_{R_{T}}$, and they have the same resistance value $R_{0}$, rendering that:

(3) $$\frac{N_{R_{T}}}{R_{0}}=\frac{4\;N_{V_{T}}}{V_{0}}.$$

The nominal parameters in our current design are that $T_{0}=298.15\;K$, $R_{0}=10\;k\Omega$, β=3500K, and $V_{0}=2\;V$. Thus, the temperature sensitivity of the bridge circuit, according to the definition, would be $S_{b}=dV_{T}/dT\approx -0.02\;V/K$. The negative value of sensitivity is a feature of using the NTC thermistor as the sensor. To achieve an equivalent noise of $0.57\;\mu K/{\mathrm{Hz}}^{1/2}$ here, the balanced bridge output noise shall satisfy $N_{V_{T}}\leq 11.4\;\mathrm{nV}/{\mathrm{Hz}}^{1/2}$, and the thermistor noise shall satisfy $N_{R_{T}}/R_{0}\leq 2.3\times 10^{-8}\;/{\mathrm{Hz}}^{1/2}$.

- Amplifier

As the temperature signal is feeble, a precision instrument amplifier (IA) can effectively improve the signal strength. The amplifier noise includes two parts [28], voltage noise $N_{e}$ and current noise $N_{i}$. At low frequencies, the equivalent input noise sources of IA (one voltage noise source and two current noise sources) are assumed to be uncorrelated with each other. By setting the gain to *G*, the amplifier output noise can be expressed as:

(4) $$N_{\mathrm{amp}}=G\;\sqrt{N_{e}^{2}+N_{i}^{2}\;\left(R_{b1}^{2}+R_{b2}^{2}\right)},$$

where $R_{b1}$ and $R_{b2}$ are the equivalent resistances of the two branches in the bridge circuit. The parameters are designed to be G=200 and $R_{b1}=R_{b2}=5\;k\Omega$. It is assumed that the voltage noise and the current noise have the same contribution. To meet the noise budget of $0.57\;\mu K/{\mathrm{Hz}}^{1/2}$, the amplifier noise must satisfy $N_{\mathrm{amp}}\leq 5.7\times 10^{-7}\;S_{b}G$, which means that the voltage noise and the current noise must satisfy $N_{e}\leq 8\;\mathrm{nV}/{\mathrm{Hz}}^{1/2}$ and $N_{i}\leq 1.1\;\mathrm{pA}/{\mathrm{Hz}}^{1/2}$, respectively.

- Filter

To effectively filter out noise outside the measurement band and improve the signal-to-noise ratio, a low-pass filter is employed. The active filter consists of an operational amplifier, which means that the filter noise is expected to be of a similar order of magnitude to the initial noise of the IA used for signal amplification. However, since the IA noise is already amplified by a factor of 200 before entering the filter, the noise contribution of the filter is anticipated to be two orders of magnitude lower than that of the IA and, therefore, negligible.

- A/D converter

The ADC is used to convert analog signals into digital signals. There are two main types of noise here, the quantization noise $N_{q}$[29] and the input-referred noise $N_{\mathrm{ref}}$ [30], which can be expressed as:

(5) $$N_{q}=\frac{V_{F}}{2^{n}\;\sqrt{6\;f_{s}}},$$

(6) $$N_{\mathrm{ref}}=\frac{V_{F}}{2^{n}\sqrt{f_{s}/2}},$$

where $V_{F}$ is the full-scale voltage, $f_{s}$ is the sampling rate, and *n* is the ADC bit size.

The ADC bit size can be designed according to the system dynamic range [31]:

(7) $$20\;\log_{10}\left(\frac{T_{\mathrm{span}}}{T_{\mathrm{res}}}\right)\leq 6.02\;n\;,$$

where $T_{\mathrm{span}}$ is the maximum range of measurement for the TMS and is approximately 4K in our design, and $T_{\mathrm{res}}$ is the desired resolution and is set to $10^{-6}$ K. Therefore, the minimum bit size required for the ADC is n=22.

The design parameters for the ADC are n=24, $V_{F}=10\;V$, and $f_{s}=5\;\mathrm{kHz}$. So the noise equivalent temperature of the ADC is $N_{\mathrm{ADC}}=\sqrt{\left(N_{q}^{2}+N_{\mathrm{ref}}^{2}\right)}/GS_{b}\approx 0.003\;\mu K/{\mathrm{Hz}}^{1/2}$, which is rather small compared with other parts.

## 3. Voltage Reference Noise Measurement System: Methods, Fabrication, and Evaluation

**Figure 2:**
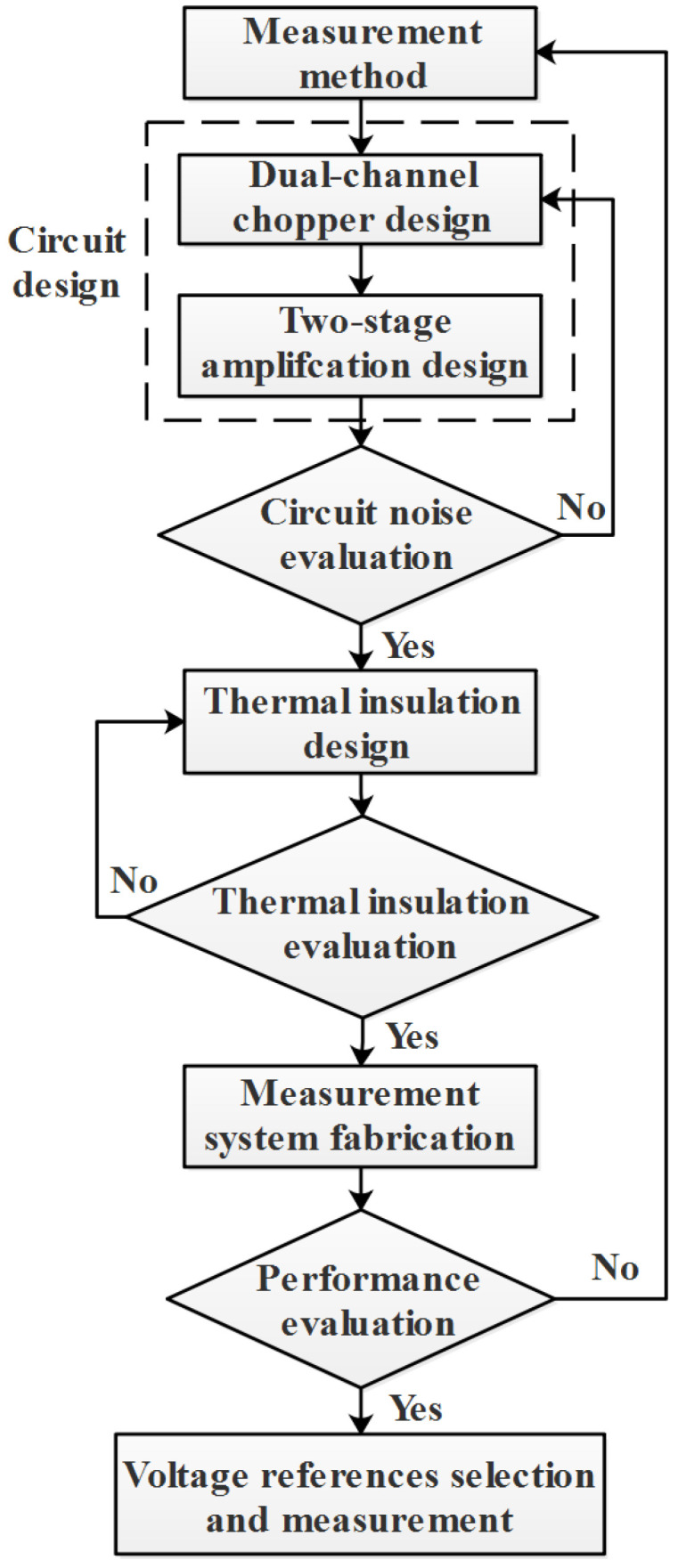
Flow chart of voltage reference noise study.

Figure 2 shows the flowchart of the VR noise study. Initially, the circuit design is developed, incorporating a dual-channel chopper and a two-stage amplification approach to improve the measurement capability of the system. Subsequently, a thermal insulation environment is devised to minimize the impact of ambient temperature during the testing process. Both the circuit and thermal insulation components undergo individual validation tests before being integrated to form a measurement system. Once the measurement system’s capability to meet the test requirements is confirmed, VR chip screening and noise measurement is performed.

### 3.1. Measurement Methods

**Figure 3:**
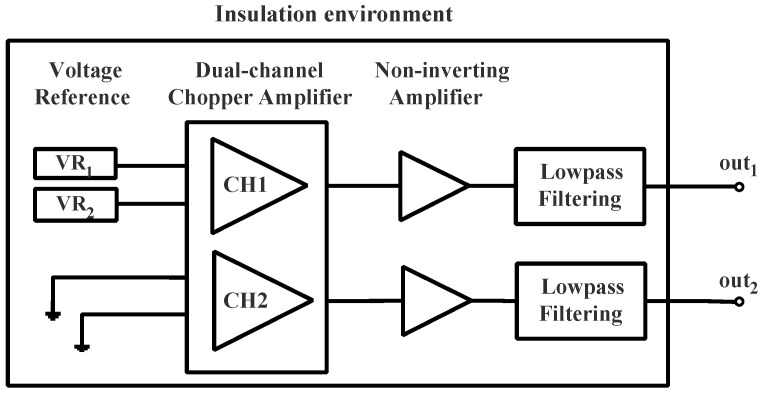
Voltage reference noise measurement scheme.

The keys to successfully measuring the VR noise at ULF include: eliminating coherent noise by performing a dual-channel measurement, reducing the 1/f noise by replacing the conventional amplifier with a chopper amplifier, improving the gain of the VR output by second-order amplification, and suppressing the temperature fluctuation by using insulation. The measurement scheme is shown in Figure 3.

#### 3.1.1. Dual-Channel Measurement Eliminates Noise Floor

The noise floor is measured independently by channel two (CH2), which has the same configuration as channel one (CH1), but its two inputs are grounded. Since the noise floor and VR noise are uncorrelated, the superposition between them is a sum-of-squares relationship. Therefore, the VR noise can be calculated from the two channel outputs.

#### 3.1.2. Chopper Suppresses 1/f Noise

Amplifying the VR outputs is necessary since the noise is too feeble to reach the detectable level. For a common-to-see amplifier, the amplification noise would increase rapidly if the frequency dropped below the corner frequency, which is about several hertz. The noise would severely affect low-frequency measurements. Chopper amplifiers are designed to avoid the 1/f noise by chopping the signals prior to the amplification. After demodulation, the signals are preserved while the 1/f of the amplification would be shifted to a high-frequency region to be the chopper noise.

#### 3.1.3. Two-Stage Amplification Improves Gain

As the chopper amplifiers cannot provide sufficient amplification gain, the non-inverting operational amplifier is utilized to boost the signals further. In one last step before the signal readout, the low-pass filters are applied to eliminate the chopper noise.

#### 3.1.4. Insulation Suppresses Temperature Fluctuation

Furthermore, the measurement can be affected by ambient temperature fluctuation. Here, a temperature coefficient of approximately 10ppm/K is estimated for the VR chips. If demanded that the temperature-induced noise is at least one order of magnitude smaller than the target signals, the maximum ambient temperature fluctuation allowed in the test is $0.1\;K/{\mathrm{Hz}}^{1/2}$. We designed a multi-layer insulation box to suppress the temperature disturbance, which is analyzed in Section 3.3.

### 3.2. Circuit Design

#### 3.2.1. Dual-Channel Chopper

The dual-channel chopper amplifier ADA4522-2 was used in the test system. The choice of the chopper amplifier was mainly based on the chopper frequency $f_{\mathrm{cp}}$ and the input noise $N_{\mathrm{in}}$. As mentioned, the low-frequency amplification noise would be shifted to the vicinity of the $f_{\mathrm{cp}}$. Thus, the chopper amplifier is required to have $f_{\mathrm{cp}}\gg 1\;\mathrm{Hz}$ to avoid interference by the 1/f noise in the measurement frequency band. In addition, it is required to have $N_{\mathrm{in}}\ll 10^{-5}\;/{\mathrm{Hz}}^{1/2}$ to prevent overlapping the VR noise. The ADA4522-2 chosen for this study meets the requirements, with a chopper frequency of 4.8MHz and an input noise level around $10^{-9}\;/{\mathrm{Hz}}^{1/2}$.

In the test, two VR chips of the same type are connected to channel one (CH1), so that the output can be expressed as:

(8) $$N_{\mathrm{out}1}=\sqrt{2\;{\left(G\;N_{\mathrm{VR}}\right)}^{2}+N_{\mathrm{misc}}^{2}}\;,$$

where $N_{\mathrm{VR}}$ is the VR noise to be assessed, and $N_{\mathrm{misc}}$ the other incoherent noise in the circuit.

Channel two (CH2) is connected to the ground, so that the output $N_{\mathrm{out}2}=N_{\mathrm{misc}}$. Then, theoretically, the VR noise can be assessed by:

(9) $$N_{\mathrm{VR}}=\sqrt{\frac{N_{\mathrm{out}1}^{2}-N_{\mathrm{out}2}^{2}}{2G^{2}}}.$$

#### 3.2.2. Two-Stage Amplification

In order to match the readout device, the digital multimeter (DMM) 3458A, the signals require a second amplification. Conventionally, the readout noise $N_{\mathrm{DMM}}$ should be at least one order of magnitude smaller than the signal, implying that $\sqrt{2}\;G\;N_{\mathrm{VR}}>10\;N_{\mathrm{DMM}}$. However, the DMM’s readout noise is dependent on its range setting. For the 3458A, it can be expressed as $N_{\mathrm{DMM}}=k_{n,R}N_{10V}$, where $N_{10V}$ represents the noise level of the digital multimeter at the 10V range, and $k_{n,R}$ represents the noise multiplier for different ranges, as shown in Table 2 [20]. In order to achieve higher resolution, the range of the digital multimeter is set to 1V. As a result, the output of the circuit designed for this purpose should not exceed the selected range, which means that the offset voltage in the circuit satisfies that $G\;V_{\mathrm{ofs}}<1\;V$.

By considering $N_{\mathrm{VR}}\approx 10^{-5}\;/{\mathrm{Hz}}^{1/2}$, $N_{\mathrm{DMM}}\approx 10^{-4}\;/{\mathrm{Hz}}^{1/2}$, and $V_{\mathrm{ofs}}\approx 100\;\mu V$, the most suitable range for the gain is found to be $10^{2}<G<10^{4}$. We use the intermediate value, that $G=10^{3}$, in the design.

**Figure 4:**
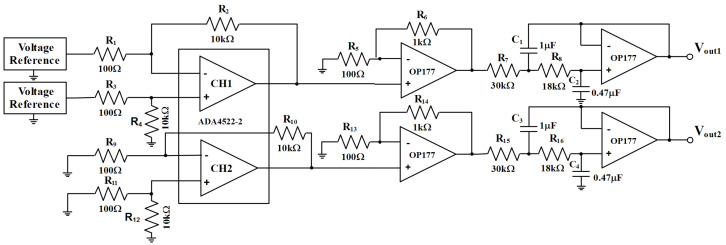
Schematic diagram of the voltage reference noise measurement circuit.

The circuit diagram of the system is shown in Figure 4. The chopper amplifier ADA4522-2 is the main amplifier, with a gain of 100 to ensure amplification of the signal while avoiding the introduction of 1/f noise. Then, the non-inverting amplifier, consisting of OP177 with a gain of 11, is used for secondary amplification. The final total gain reaches 1100. The use of an operational amplifier (OP177) for the second stage of amplification is due to the potential for intermodulation distortion when using two chopper amplifiers. The signal is expected to be $1\;\mathrm{mV}/{\mathrm{Hz}}^{1/2}$ after 100 times amplification by the chopper amplifier in the first stage, which is already strong enough to overcome the $1\;\mu V/{\mathrm{Hz}}^{1/2}$ level of noise at the 0.1mHz frequency of the OP177. Before the output, a second-order low-pass filter with a cutoff frequency of 10 Hz is used to eliminate the chopping frequency of 4.8MHz. CH2 is configured the same as CH1, except that the input to CH2 is grounded to obtain the system noise floor.

### 3.3. Thermal Insulation

**Figure 5:**
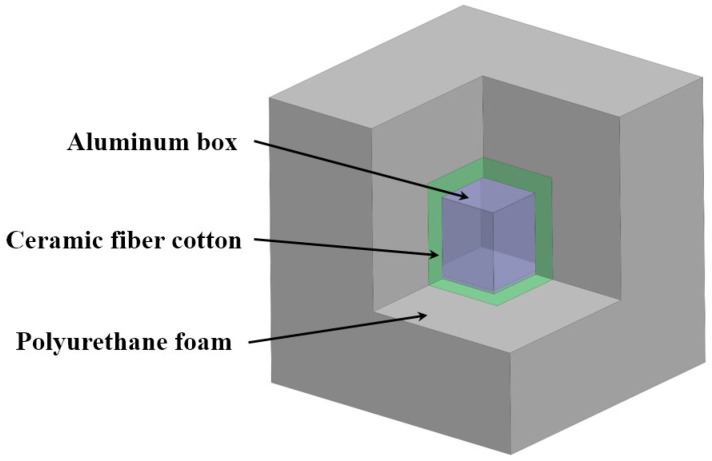
Structure diagram of insulation box.

To suppress temperature fluctuations, we designed and prepared an assembled box with a multi-layer insulation structure, as shown in Figure 5. The outer layer is made of polyurethane foam, which has low conductivity, to thermally insulate the interiors from the indoor environment. The middle layer of the assembly box is filled with ceramic fiber cotton to reduce heat leakage. The inner layer is a metal aluminum box that houses the measurement circuit. The aluminum box can equalize the internal temperature. The material parameters of each layer can be found in Table 3.

**Figure 6:**
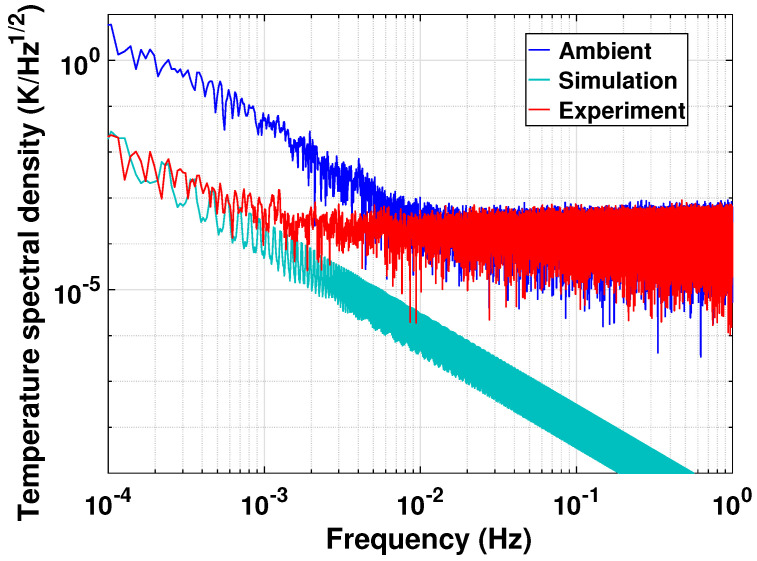
Temperature spectral density of insulation box. The blue curve shows the exterior ambient temperature, and the cyan and red curves show the simulated and experimental results of the interior temperature of the insulation box, respectively.

The insulation performance of the box is evaluated using both finite element method (FEM) simulation and temperature measurement. The results are shown in Figure 6. Temperature measurements are carried out simultaneously on both the exterior and interior surfaces of the box. Then, the transient FEM simulation is conducted to derive a theoretical fluctuation inside, by using the temperature data measured on the exterior surface as disturbance. The materials data used in the simulation are listed in Table 3.

The spectral density indicates that the temperature fluctuation is effectively suppressed to $0.1\;K/{\mathrm{Hz}}^{1/2}$ in the frequency band of 0.1 mHz–1 Hz. The interior temperature data above 1mHz are actually overlapped by the detector’s noise floor of ∼$10^{-4}\;K/{\mathrm{Hz}}^{1/2}$. The simulation shows the tendency that the temperature fluctuations decrease rapidly with frequency, and that the interior temperature is more stable than the measurement at frequencies above 1mHz.

### 3.4. System Setup and Performance Assessment

**Figure 7:**
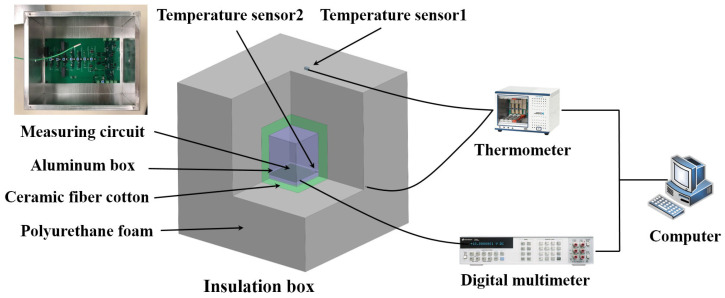
Voltage reference noise measurement system.

The devices were placed and connected properly to build the measurement system, as shown in Figure 7. In addition to the measurement circuit, the system included a digital multimeter 3458A and a thermometer PXIe-4357, two temperature sensors PT100, and a computer for data recording and processing. A photograph of the actual setup of the measurement system is available in the supplementary material.

Prior to evaluating the system’s low-frequency performance, a preliminary validation was conducted on the system within a frequency range of 100–1000 Hz, with the low-pass filter deactivated. This was carried out to ascertain the system’s operational reliability by comparing the outputs to the official noise data provided by most VR chips within the frequency range. In addition, the coherence of the CH1 and CH2 channels’ outputs was examined during the validation process.

**Figure 8:**
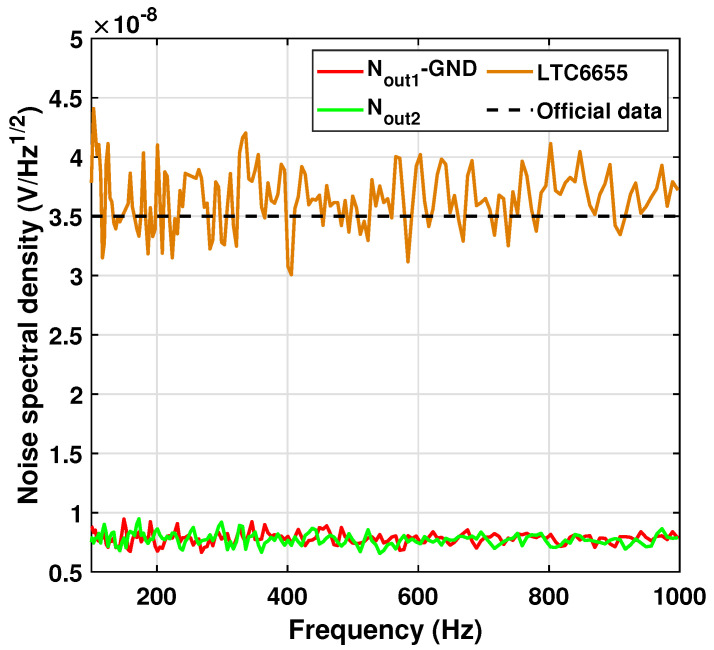
System validation in common frequency range.

To conduct the validation, both channels were grounded to check the noise floor and coherency, as shown by the red and green lines in Figure 8. The results revealed that the noise floors were significantly lower than the expected noise of the VR chips, and the channels were highly coherent with each other. Therefore, the precondition of applying dual-channel cancellation, as described in Equation (9), was deemed justified.

Then, the system was connected to the VR chip LTC6655, which has the lowest official noise, to continue the validation. As shown in Figure 8, the orange line represents the processed result obtained using the dual-channel cancellation method. The result shows the same level of noise as the official data, which justifies the validation of the method.

The low-frequency assessment has a procedure similar to the validation, but this time we focus on the much lower frequency. Firstly, the DMM inputs are short-circuited to measure the noise floor. Then, they are connected to CH2 to record the circuit noise. Each measurement is performed for at least 24 h to acquire low-frequency information. In the data processing, we obtain the assessed noise by dividing the raw outputs by the system gain. As the last step, we perform amplitude spectral analysis on the noise and divide the derived spectra by the nominal voltage of 2.5V to obtain the normalized noise.

**Figure 9:**
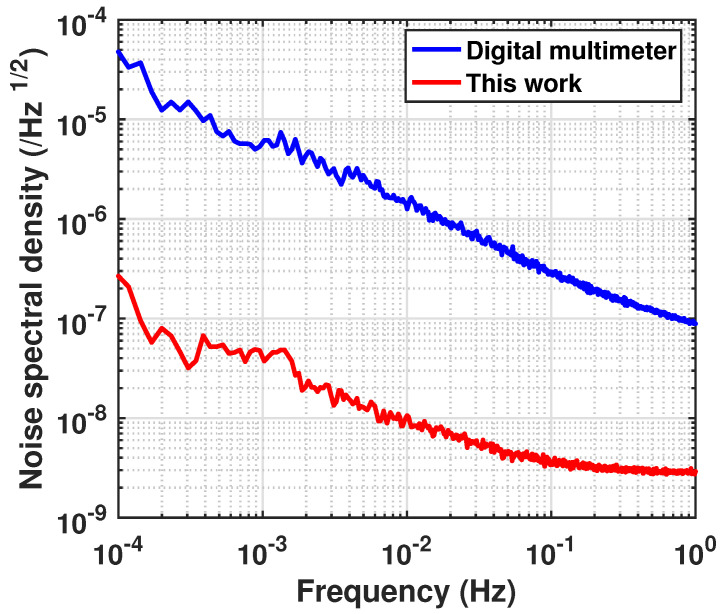
Digital multimeter noise and the equivalent input noise of this work.

To demonstrate the functionality of the amplification circuit, we present a comparison between the minimal noise floor of the DMM and the detection capability of the complete test system (the noise of CH2) in Figure 9. With the circuit, the detection capability at 0.1mHz has been improved by two orders of magnitude, to $3\times 10^{-7}\;/{\mathrm{Hz}}^{1/2}$, compared with that of the bare-connected DMM, which is of $4\times 10^{-5}\;/{\mathrm{Hz}}^{1/2}$.

After completing the system performance evaluation, CH1 is used to acquire the output data that contains the VR chip noise during the ULF measurement. CH2 is then used to acquire output data that contains only the system noise floor. The VR noise is calculated from the noise spectral density of both channels using Equation (9). The normalized VR chip noise is obtained by dividing the VR noise by the rated voltage.

## 4. Voltage References Selection

Most manufacturers prefer to document the peak-to-peak noise information about their chips in the frequency range of 0.1–10 Hz. In the absence of special needs, no attention is paid to their noise characteristics in the frequency band below 0.1 Hz. The noise characteristics in this frequency band need to be tested by the user.

Before the measurement, it is assumed that VR chips with low peak-to-peak noise in the 0.1–10 Hz band also have low noise characteristics at lower frequencies. We selected seven models of VR chips which have the smallest on-document noise, from hundreds of others, as shown in Table 4. They are the ADR441, the ADR4525, the LTC6655, the MAX6126, the MAX6226, the MAX6325, and the MAX6071. Please note that the selected models for the test correspond to the top seven on the list, where the rest of the models are demonstrated for comparison.

## 5. Results of Voltage Reference Noise

**Figure 10:**
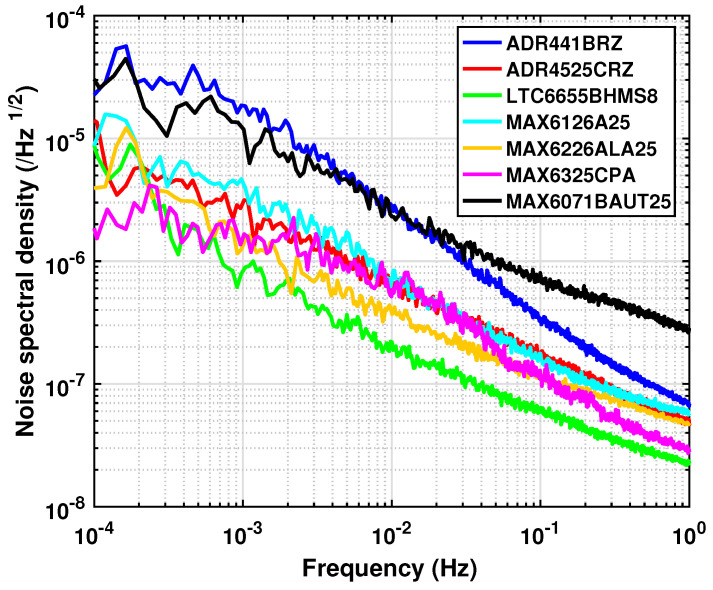
Noise of the seven voltage references in the frequency range of 1 mHz–1 Hz.

The normalized noise for the seven selected models of VR chip are measured by the system, as shown in Figure 10. The noise spectra in the ULF band demonstrate a frequency feature similar to the standard 1/f noise. For the first time, we are able to measure the kind of noise down to a frequency of 0.1mHz.

As an important result, there are five of the seven models being tested that can meet the requirements of the TMS’s development. They are the ADR4525, the LTC6655, the MAX6126, the MAX6226, and the MAX6325. At the frequency of 0.1mHz, they have noise of the same order of magnitude of ∼$5\times 10^{-6}\;/{\mathrm{Hz}}^{1/2}$, except for the MAX6325 which has the smallest noise, of $2\times 10^{-6}\;/{\mathrm{Hz}}^{1/2}$. Meanwhile, in the frequency range of 1 mHz–1 Hz, the LTC6655 is found to be the most stable instead.

The ADR441 and the MAX6071 are the two models with the highest noise, at 0.1mHz. This noise exceeds the requirement of $2.8\times 10^{-5}\;/{\mathrm{Hz}}^{1/2}$. Therefore, the two models are not suitable for being used in the TMS. Moreover, their noise in the frequency range of 0.1–10 mHz is so close, although their parameters given in documents are completely different. This indicates that the documented noise, generally for a frequency of around 1Hz, cannot represent the noise in the ULF band. The VR chip noise can be vastly different with the frequency change.

At the frequency of 1Hz, the noise levels of the four tested chips (ADR441, ADR4525, MAX6126, and MAX6226) were found to be similar. However, when the frequency decreases to ∼$10^{-2}\;\mathrm{Hz}$, the ADR441, which is of the XFET-arch, exhibited much higher noise levels than the other chips in the test.

Based on the test results, it can be concluded that the noise characteristics of VR chips in the ULF band are complex and cannot be simply predicted by the peak-to-peak noise specified in the datasheet. To develop the TMS, testing the VR chip candidates in the designated frequency range is an ineluctable step.

## 6. Conclusions

This study presents the development of a dedicated system for measuring ultra-low-frequency noise of voltage reference chips, which are critical components in various precision electrical devices. The system is designed based on the dual-channel chopper amplification principle, which effectively mitigates the impact of 1/f noise and noise floor. Furthermore, a multi-layer thermal insulation box is utilized to minimize low-frequency ambient temperature fluctuations. As a result, our system has achieved a normalized detection capability of $3\times 10^{-7}\;/{\mathrm{Hz}}^{1/2}\;@\;0.1\;\mathrm{mHz}$.

The noise spectral density data of seven models of voltage reference chips are obtained in the frequency range of 0.1 mHz–1 Hz. As part of a project to develop a precision temperature measurement subsystem for space gravitational wave detectors, the test results have confirmed five models of voltage reference chips applicable for future development. The findings of this study demonstrate that ultra-low-frequency noise is a complex issue. It is essential for users to pay closer attention to this issue to ensure optimal performance of precision electrical systems that rely on these components.

## Figures and Tables

**Table 1:** Noise budget for the temperature measurement subsystem.

Noise Source	Budget ($\mu K/{\mathrm{Hz}}^{1/2}$)	Requirements (0.1 mHz–1 Hz)
Voltage reference	0.57	$N_{V}/V_{0}\leq 2.8\times 10^{-5}\;/{\mathrm{Hz}}^{1/2}$
Thermistor	0.57	$N_{R_{T}}/R_{0}\leq 2.3\times 10^{-8}\;/{\mathrm{Hz}}^{1/2}$
Amplifier	0.57	$N_{e}\leq 8\;\mathrm{nV}/{\mathrm{Hz}}^{1/2},$
		$N_{i}\leq 1.1\;\mathrm{pA}/{\mathrm{Hz}}^{1/2}.$
Others	0.16	−
Total	1.0	−

**Table 2:** DC voltage range setting and noise multiplier of the multimeter 3458A [20].

Range	$k_{n,R}$
0.1V	0.2 (0.07)
1V	0.2
10V	1
100V	20
1000V	100

**Table 3:** Parameters of the insulation box.

Layer	Material	Conductivity [W/(m·K)]	Specific Heat [J/(kg·K)]	Density [$\mathrm{kg}/m^{3}$]	Thickness [cm]
Outer	Polyurethane foam	0.02	1000	46	20
Middle	Ceramic fiber cotton	0.08	1000	300	5
Inner	Aluminum	900	237	2700	0.5

**Table 4:** Voltage reference data given by the manufacturers [32,33,34].

Order	Model	Manufacturer	Type	Noise in 0.1–10 Hz ($\mu V_{p-p}$)	Temperature Coefficient (ppm/K)
1	ADR441BRZ	Analog Devices	XFET	1.2	1
2	ADR4525CRZ	Analog Devices	Bandgap	1.25	1
3	LTC6655BHMS8	Analog Devices	Bandgap	0.625	2
4	MAX6126A25	Maxim Integrated	Proprietary	1.45	1
5	MAX6226ALA25	Maxim Integrated	Proprietary	1.45	1
6	MAX6325CPA	Maxim Integrated	Buried zenner	1.5	0.5
7	MAX6071BAUT25	Maxim Integrated	Bandgap	4.8	1.5
8	ADR291WF	Analog Devices	XFET	8	5
9	REF03GS	Analog Devices	Bandgap	6	10
10	REF192GR	Analog Devices	Bandgap	25	10
11	LT1034B	Analog Devices	Bandgap	6	10
12	LT1461B	Analog Devices	Bandgap	20	3
13	MAX6025A	Maxim Integrated	Bandgap	50	6
14	MAX6033B	Maxim Integrated	Bandgap	16	1.5
15	MAX6125E	Maxim Integrated	−	15	15
16	MAX6192A	Maxim Integrated	Bandgap	60	2
17	REF3225A	Texas Instruments	Bandgap	33	4
18	REF3425I	Texas Instruments	Bandgap	12.5	2.5
19	REF4132A	Texas Instruments	Bandgap	37.5	12
20	REF6025I	Texas Instruments	Bandgap	7.5	5

## Data Availability

The data presented in this study are available on request from the corresponding author.

## Supplementary Materials

The following supporting information can be downloaded at: https://www.mdpi.com/article/10.3390/s23104611/s1, Figure S1. Measurement setup with the insulation box open. Figure S2. Measurement setup with the insulation box closed.
