# Supplementary material for: Study on the Voltage Reference Noise at Sub-Millihertz Frequencies for Developing an Ultra-Stable Temperature Measurement Subsystem
Source: Sensors (Basel). 2023 May 10;23(10):4611. doi: 10.3390/s23104611 (PMC10221231; doi:10.3390/s23104611)
Supplement: Supplementary file 1 [file sensors-23-04611-s001.zip › sensors-2296695-supplementary.pdf]

Actual setup figure of the measurement system

Figure S1 shows the thermal insulation box in an open state, with the measurement circuit inside. The digital multimeter measures the output of the circuit, and data is collected via the computer. Two thermometer sensors monitor the temperature inside and outside the thermal insulation box during the measurement. Figure S2 shows the thermal insulation box in a closed state, with the top filled with a polyurethane foam board that fills the gap.

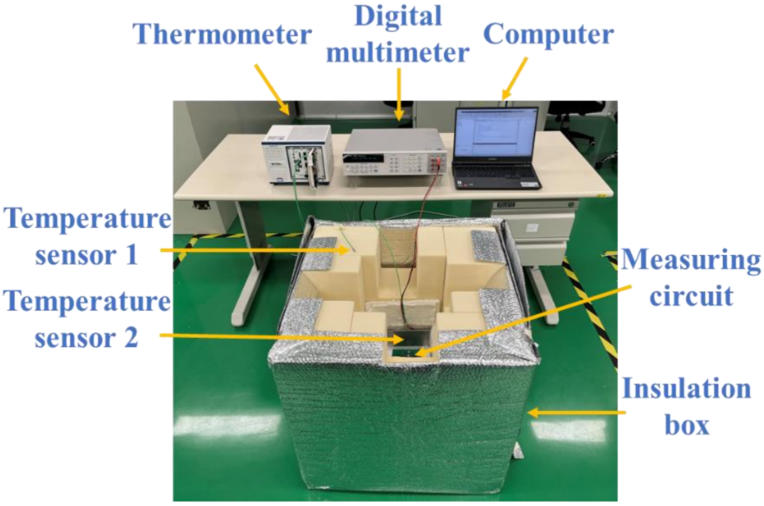

Figure S1. Measurement setup with the insulation box open.

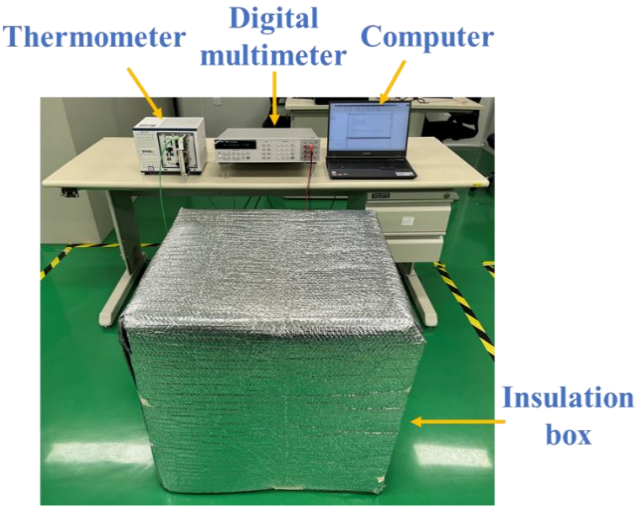

Figure S2. Measurement setup with the insulation box closed.
